# Meeting of the Medical Association of Georgia

**Published:** 1893-04

**Authors:** A. A. Smith, Dan H. Howell

**Affiliations:** President; Secretary


### MEETING OF THE ASSOCIATION.

The programme of the forty-fourth Annual Session of the Medical Association of Georgia to be held in Americus, Ga., April 17, 20 and 21, shows that we are to have an intellectual treat rarely furnished even by the members of the profession of the Empire State of the South.

The officers of the Association are working men as well as active practitioners. They will do their duty. Men from all portions of the State will report the results of their labor. They will meet in social gatherings and renew old acquaintances and form new ties. We anticipate great good to come from this meeting.

PAPERS TO BE READ.

The President's Annual Address, A. A. Smith, M. D., Hawkinsville.

Orator's Address — Woman's Relations to the Practice of Medicine, Frank Ridley, M. D., LaGrange.

Diffuse Tranmatic Aneurism, of Anterior Tibial Artery—Ligation of Femoral, F. R. Calhoun, M. D., Cartersville.

Multiple Neuritis "Alcoholic," Mark H. O'Daniel, M. D., Macon

Extra Uterine Pregnancy, R. R. Kime, M. D., Atlanta.

Puerperal Eclampsia, with Special Reference to its Cause and Treatment, A. C. Davidson, M. D., Sharon.

Asphyxia Neophytorum, R. J. Nunn, M. D., Savannah.

Stab of the Stomach—The Organ Protruding Through Abdominal Wall—Laparotomy—Recovery, J. W. Griggs, M. D., West Point.

Operation for Fistula in Ano by Ligation, John Hill, M. D., Washington.

The Contagiousness of Consumption, J. G. Hopkins, M. D., Thomasville

The Disappointment of the Menopause, J. C. Avary, M. D., Atlanta.

Headache Versus Glaucoma, W. T. Bullard, M. D., Columbus.

"Shot Gun" Prescriptions, C. C. Hart, M. D., Cross Keys.

A Review of "Dr. Senns' Views on Elastic Constructions," W. H. Elliot, M. D., Savannah.

The Practice of Medicine in Georgia, A. C. Blain, M. D., Macon.

Hernia, W. F. Westmoreland, M. D., Atlanta.

Anti-Pyretics, Translated from the German, of a Paper Read Before the National Medical Congress in Berlin, by Cantani—Translation made by Myself, S. B. Poland, M. D., Griswoldville.

Science in Medicine and Surgery, J. McFadden Gaston, M. D., Atlanta.

Ophthalmia of the New Born, S. Latimer Phillips, M. D., Savannah.

Periproctitis with an Abscess, and Report of a Case, M. L. Currie, M. D., Mt. Vernon.

The Necessity for a Medical Examining Board in Georgia, L. B. Grandy, M. D., Atlanta.

State and Municipal Hygiene, J. C. Avery, M. D., Atlanta.

Rare Case in Obstetric Practice, O. H. Buford, M. D., Cartersville.

Sterility in the Male, C. Evans Johnson, Atlanta.

Conditions Indicating Abdominal Operations and Report of Cases, Frank M. Ridley, M. D., LaGrange.

Pneumonia, O. T. Kenyon, M. D., Weston.

The Function and Nutritive Value of Foods, Louis H. Jones, Atlanta.

Stone in the Bladder, with Report of Cases, F. W. McRae, M. D., Atlanta.

Non Surgical Treatment of Typhilitis, E. H. Richardson, M. D., Atlanta.

Drainage in Pelvic Surgery, Geo. H. Noble, M. D,, Atlanta.

Specialism in Medicine, A. S. Hawes, M. D., Atlanta.

Partial Tenotomy a Radical Cure for Heterophoralgia, C. H. Peete, M. D., Macon.

Pathology of Gynecic Neuroses, Ross P. Cox, M. D., Rome.

Persistent Remittant or so-called Typho-Malarial Fever, with Report of Cases, W. P. Williams, M. D., Blackshear.

Some Remarks on Aseptic Surgery, with Exhibition of Sterilizing Methods, T. M. McIntosh, M. D., Thomasville.

Mechanical Treatment of Some Skin Anomalies, M. B. Hutchins, M. D., Atlanta.

Four Women who Refused Oophorectomy and their Subsequent Histories, H. McHatton, M. D., Macon.

Toxic Amblyopia, by James H. Shorter, M. D., Macon.

Symptoms Indicating Abnormal Conditions of the Alimentary Canal, with Report of Cases, by C. D. Hunt, M. D., Atlanta.

Typhoid Fever, by N. M. Darden, M. D., Norwood.

The Treatment of Some of the Most Common Forms of Pelvic Congestion in the Female, by W. A. Crow, M. D., West End.

Puerpal Eclampsia, with Report of Cases, by J. M. Head, M. D., Zebulon.

Fracture of the Skull, with Report of Cases, by W. R. Googe, M. D., Abbeville.

Puerpal Septicæmia and its Treatment, by J. J. Darby, M. D., Americus.

Ante and Retro-flexions of the Uterus, their Pathology and Treatment, by W. W. Stewart, M. D., Columbus.

Two Rare Cases in Practice, by P. L. Hillsman, M. D., Albany.

Cases of Perityphilitic Abscess, by C. J. Carter, M. D., Lake Park.

A Case of Aneurism of the Thoracic Aorta, by N. P. Jelks, M. D., Hawkinsville.

Injury to Foot, Ligation of Anterior and Posterior Tibial Arteries, by W. B. Tate, M. D., Tate.

A. A. SMITH, M. D., President.

DAN H. HOWELL, M. D., Secretary.

